# The Association of Arsenic Metabolism and Blood Pressure: A Cross-Sectional Analysis in the MesoAmerican Nephropathy Occupational Study (MANOS)

**DOI:** 10.21203/rs.3.rs-9107335/v1

**Published:** 2026-03-24

**Authors:** Margaret Quaid, Kathryn Rodgers, Juan Jose Amador Velázquez, Ramón García-Trabanino, Emmanuel Jarquin, Damaris Lopez-Pilarte, Jessica Leibler, Daniel Brooks, Ronald A Glabonjat, Ana Navas-Acien, Maria Argos, Madeleine K. Scammell

**Affiliations:** Boston University School of Public Health; Boston University School of Public Health; Boston University School of Public Health; Emergency Social Fund for Health; Agencia para el Desarrollo y la Salud Agropecuaria (AGDYSA); Boston University School of Public Health; Boston University School of Public Health; Boston University School of Public Health; Columbia University, Mailman School of Public Health; Columbia University, Mailman School of Public Health; Boston University School of Public Health; Boston University School of Public Health

**Keywords:** Arsenic, Blood pressure, Central America, Metabolism, Methylation, Speciation

## Abstract

**Background:**

Growing evidence indicates that arsenic metabolism is associated with cardiometabolic outcomes but few studies have investigated the association of arsenic metabolism with blood pressure outcomes.

**Methods:**

We evaluated cross-sectional associations between urinary arsenic metabolites and blood pressure outcomes—systolic and diastolic blood pressure, pulse pressure, and mean arterial pressure—among 393 participants in the MesoAmerican Nephropathy Occupational Study (MANOS) in El Salvador and Nicaragua. We applied three modeling approaches: (1) conventional models assessing each urinary arsenic species [inorganic arsenic (InAs), monomethylated arsenic (MMA), and dimethylated arsenic (DMA)] individually as a percentage of the sum of inorganic and methylated arsenic; (2) leave-one-out models evaluating the relative effects of two species while holding the third constant; and (3) principal components analysis (PCA) representing methylation steps of arsenic metabolism.

**Results:**

In conventional models adjusted for age, body mass index, worksite, pesticide use, smoking status, and water consumption, participants with higher vs. lower DMA% (>77.51% vs. ≤71.28% DMA over the sum of inorganic and methylated arsenic species) showed higher systolic blood pressure (β = 3.75 mmHg; 95% CI: 0.65, 6.85) and pulse pressure (β = 2.57 mmHg; 95% CI: 0.04, 5.10), while participants with higher vs. lower MMA% (>16.07% vs. ≤12.39%) showed lower systolic blood pressure (β = −3.70 mmHg; 95% CI: −6.86, −0.55) and pulse pressure (β = −2.76 mmHg; 95% CI: −5.33, −0.19). In leave-one-out models, higher DMA% (>77.51% vs. <71.28%) as a result of lower MMA%, was associated with higher systolic blood pressure (β = 7.24 mmHg; 95% CI: 2.25, 12.2), pulse pressure (β = 5.29 mmHg; 95% CI: 1.22, 9.36), and mean arterial pressure (β = 3.71 mmHg; 95% CI: −0.08, 7.50). PCA results supported these findings. The second methylation step from MMA to DMA was associated with higher systolic blood pressure (β = 0.93 mmHg; 95% CI: 0.11, 1.75) and pulse pressure (β = 0.74 mmHg; 95% CI: 0.07, 1.40).

**Conclusions:**

Our findings suggest that biomarkers of efficient methylation of inorganic arsenic to DMA are associated with higher blood pressure compared to partial methylation to MMA, highlighting the importance of arsenic metabolism profiles in cardiovascular risk assessment.

## Background

Relative to the global average, people who live in Central America have high rates of a number of adverse health conditions, including hypertension and chronic kidney disease (1–3). Researchers have investigated several potential risk factors in the region, including the widespread current and historical use of pesticides, the intense concentration of sugarcane production, and the prevalence of toxic elements like arsenic in drinking water (4–8). Arsenic exposure has been linked with adverse cardiovascular outcomes, including hypertension, in populations in the United States, Europe, China, and Bangladesh; however, the relationship of arsenic exposure and arsenic metabolism has not been studied in Central American populations (9–11).

Arsenic contamination in environmental media is highly prevalent in many countries in Central America, including El Salvador and Nicaragua (5, 12, 13). This contamination has been found in the groundwater of El Salvador and in the municipal drinking water of Nicaragua at levels which frequently exceed the World Health Organization’s 10 μg/L guideline for arsenic in drinking water (12–14).

When introduced to the body, inorganic arsenic (InAs) is metabolized primarily in the liver, facilitating its elimination (15). This metabolism occurs through a series of reduction and methylation steps, with arsenic-3-methyltransferase (*AS3MT*) methylating trivalent arsenic species using S-adenosyl methionine (SAM) as the methyl donor (16). In brief, arsenite (InAs^3+^) is methylated to monomethylarsonic acid (MMA^5+^), reduced to monomethylarsonous acid (MMA^3+^), and then subsequently methylated to dimethylarsinic acid (DMA^5+^), which can be reduced to dimethylarsinous acid (DMA^3+^). The analysis presented in this study does not differentiate between the oxidation states of arsenic species. The proportion of arsenic metabolites in the urine acts as a proxy of arsenic metabolism efficiency (17).

This study investigates the relationship between arsenic exposure (speciated and sum of species) and arsenic metabolism (measured as the percentage of each species over the sum of inorganic and methylated arsenic species: InAs%, MMA%, and DMA%) with systolic and diastolic blood pressure, pulse pressure, and mean arterial pressure in an occupational cohort in Central America. This study includes participants from El Salvador and Nicaragua who are part of the MesoAmerican Nephropathy Occupational Study (MANOS), which was designed to investigate risk factors for the high rates of chronic kidney disease of unknown etiology in the region (18).

## Methods

### Study Population and Data Collection

The MesoAmerican Nephropathy Occupational Study (MANOS) cohort has been previously described (18). Briefly, 569 males, aged 18 to 45 years, were enrolled at their worksites between January and May of 2018 in El Salvador and Nicaragua. Known hypertension was an exclusion criterion: participants were ineligible if they reported hypertension medication use or if their blood pressure on enrollment was higher than 160/95 mmHg. Data were collected on each participant over the three workdays following enrollment.

Before urine was analyzed for arsenic speciation, all participants’ serum was analyzed for creatinine, which was used to calculate estimated glomerular filtration rate (eGFR) using the Chronic Kidney Disease Epidemiology Collaboration for males (18, 19). Only those with an eGFR > 45 mL/min/1.73 m2 were selected for total urinary arsenic quantification to minimize the risk that severely low kidney function would impact the concentration of arsenic in urine (20). Of the participants who had an eGFR > 45 mL/min/1.73 m2, only those with urinary total arsenic concentrations >5 μg/L were selected for speciation analysis. This resulted in a cohort of 404 participants (71% of all MANOS participants) with speciated arsenic measurements. An additional four participants were missing blood pressure measures, and seven participants were missing data on body mass index (BMI) or water consumption. The final sample available for this analysis consisted of 393 participants (69%). All participants provided written consent (18).

### Arsenic Quantification and Urine Osmolality

Spot urine samples were collected in the field before work on the third day of data collection and transferred to a nearby laboratory for aliquoting and on-site analyses. Urine osmolality was measured via a handheld refractometer and used as a covariate to adjust for urine dilution. Urine samples were then stored at ¬¬¬ 80°C in each country before being shipped on dry ice to the Boston University School of Public Health for long-term storage and distribution to the analytical laboratories (18).

Arsenic speciation was conducted at the Multi-Element Trace Analysis Laboratory (METALab) at Columbia University Mailman School of Public Health (21). The samples were thawed, and an aliquot (100 μL) was treated with hydrogen peroxide (30%wt, 10 μL) solution by volume. The resulting solutions were heated to 60°C for 30 minutes. The solution was diluted with mobile phase (390 μL) before injection on a PRP-X100 high-performance liquid chromatography column for separation. InAs, MMA, and DMA were quantified using inductively-coupled plasma mass spectrometry with oxygen as a reaction gas. This method converts all arsenic species to a 5+ oxidation state, and therefore it does not distinguish between oxidation states within the arsenic species. Full details can be found in Glabonjat et al. (21). For quality control, the speciated arsenic samples were corrected by calibration background, instrumental drift, and method blanks. The method blanks were run alongside the urine samples and were prepared in the same manner to quantify any potential contamination in the sample. The standard deviation of the measured blanks was then multiplied by 3.33 to determine the method detection limit (MDL) (21). The MDL for InAs was 0.05 μg/L, that of MMA was 0.04 μg/L, and that of DMA was 0.03 μg/L. Any arsenic concentrations below the MDL were imputed with MDL⁄√2. All arsenic species were detected in 100% of participants, apart from InAs, which was detected in 98.8% of participants.

Total arsenic was calculated as the sum of InAs, MMA and DMA. The percentage of urinary arsenic species were calculated by the following equations:

(Equation 1) InAs%=(InAsconcentration)/(Totalarsenicconcentration)×100%

(Equation 2) MMA%=(MMAconcentration)/(Totalarsenicconcentration)×100%

(Equation 3) DMA%=(DMAconcentration)/(Totalarsenicconcentration)×100%

The percentages of InAs, MMA, and DMA and the absolute concentrations of InAs, MMA, and DMA, and total arsenic were divided into tertiles of the exposure distributions for statistical modeling, with the lowest tertile of exposure used as the reference.

### Blood Pressure Measurement and Calculation

Blood pressure was directly measured on the third day of data collection, at the same time urine was collected, before the work shift to represent the resting blood pressure, unaffected by work strain. A single reading of seated systolic and diastolic blood pressure was taken by trained clinicians using an Omron^®^ automatic blood pressure cuff (Omron Healthcare, Kyoto, Japan). Pulse pressure was calculated by subtracting the diastolic blood pressure from the systolic blood pressure. Previous studies have identified pulse pressure as a relevant cardiovascular health metric and shown that heightened pulse pressure is associated with adverse health outcomes (22, 23). Mean arterial pressure (MAP) was calculated using the following equation (24):

(Equation 4) MAP=diastolicbloodpressure+(1/3)×pulsepressure

Increased MAP is associated with adverse cardiovascular outcomes (24, 25).

### Covariates

Trained research staff conducted extensive initial interviews, as well as pre-work shift and post-work shift interviews with each study participant for the first three days of the study. Data on industry, job, age, current smoking status (yes/no), and current alcohol consumption (yes/no) were collected at initial interview. Data on work activities, pesticide use at work (yes/no), water consumption, and non-water liquid consumption, were collected from post-shift interview. Pre-work shift measures of height and weight on the first day were used to determine BMI for each participant.

### Statistical Analysis

We used linear regression to evaluate associations between arsenic exposure and arsenic metabolism with four blood pressure traits: systolic blood pressure, diastolic blood pressure, pulse pressure, and MAP. In partially adjusted models (Model 1), we adjusted for age, BMI, and worksite (which captures information on both country and industry of work). In fully adjusted models (Model 2), we further adjusted for pesticide use in the past three days (yes, no), current smoking status (yes, no), and water consumption at work (liters on day of interview). We first evaluated the relationship between tertiles of urinary arsenic exposure by concentration (InAs concentration, MMA concentration, DMA concentration, and the summed concentrations of InAs, MMA, and DMA) separately with each of the four blood pressure outcomes using linear regression, adjusting for the covariates listed above, as well as urinary osmolality to account for differences in urine dilution.

Beyond arsenic exposure, we evaluated biomarkers of arsenic metabolism. Because the proportions of urinary arsenic species are interdependent, interpreting associations for individual species is challenging (26). To address this, we applied three complementary modeling strategies, as has been done in the prior literature (26–29). The first method was conventional modeling, in which each arsenic species was modeled separately as a percentage of each species over the sum of inorganic and methylated arsenic in urine to estimate its individual association with blood pressure traits. The second method was the leave-one-out model, in which two of the three arsenic species were included in each model, allowing us to interpret the effect of increasing one species while holding another constant. The third strategy was principal components analysis (PCA) in which two PCs were derived, which represent the first methylation step (InAs to MMA) and the second methylation step (MMA to DMA). The resulting PCs were separately modeled as biomarkers of arsenic metabolism.

To examine the risk of potential confounding by seafood intake, which can be an additional source of DMA and arsenobetaine exposure, we report the Spearman correlation between DMA% and arsenobetaine levels. In sensitivity analyses, we evaluated (1) the inclusion of the sum of organic and methylated arsenic species in the modeling, (2) the inclusion of arsenobetaine in the modeling, and (3) the additional inclusion of alcohol consumption as a covariate. We additionally used an interaction term to investigate the possibility of effect modification by eGFR because kidney function can be related to both the level of arsenic metabolites excreted in the urine and to blood pressure (30, 31).

## Results

In total, 182 participants from El Salvador and 211 participants from Nicaragua were included in these analyses. Characteristics of the study sample are summarized in Table 1. Participants were, on average, 28 years old, had a BMI of 24 kg/m^2^, and approximately 40% were current smokers. The individuals included in this study do not meaningfully differ from the full cohort on any investigated covariates (Table 1). Participant characteristics by exposure status is summarized in Supplementary Table 1 and there appear to be differences in exposure level by BMI and worksite.

In urinary arsenic concentration models using the sum of inorganic and methylated arsenic species, the second tertile of exposure (particularly for InAs and MMA) was negatively associated with systolic blood pressure, diastolic blood pressure, and mean arterial pressure, though none of these relationships were observed in the highest tertile of urinary arsenic exposure (for any of the As metabolites) and the trend was not significant (Table 2).

We then assessed the associations between the percentage of each urinary arsenic species (InAs%, MMA%, and DMA%), divided into tertiles, and the four blood pressure outcomes using linear regression models (Table 3). A higher percentage of urinary DMA was positively associated with systolic blood pressure, pulse pressure, and mean arterial pressure with significant or near-significant trends. Conversely, a higher percentage of MMA was negatively associated with systolic blood pressure and pulse pressure, with significant trends. No associations were observed between InAs percentage and any of the blood pressure traits.

In the fully adjusted leave-one-out model (Table 4), a higher percentage of urinary DMA was positively associated with systolic blood pressure, pulse pressure, and mean arterial pressure when the percentage of InAs was held constant (which is interpreted as an increase in DMA% with a corresponding decrease in MMA%). These associations demonstrated a significant trend across tertiles of DMA exposure for systolic blood pressure and pulse pressure, and near-significant trends for mean arterial pressure. There is a moderate positive relationship between percentage of urinary InAs and systolic blood pressure and pulse pressure when DMA percentage is held constant (which is interpreted as an increase in InAs% corresponding to a decrease in MMA%). A higher percentage of MMA was negatively associated with systolic blood pressure, pulse pressure, and mean arterial pressure when InAs was held constant (corresponding to a decrease in DMA%), with significant trends for systolic blood pressure and pulse pressure across MMA tertiles.

Principal components analysis (PCA) was used to capture overall patterns in arsenic metabolism. The loadings of each arsenic species on the first two principal components are presented in Table 5. Based on these loadings, the components were interpreted as follows: (1) principal component 1 (PC1) primarily reflects the second methylation step, representing the conversion of MMA to DMA, supported by a strong positive loading for DMA and inverse loadings for MMA and InAs; and (2) principal component 2 (PC2) reflects the first methylation step, representing the conversion from InAs to MMA, as indicated by inverse loadings between InAs and MMA. In fully adjusted models, PC1 was positively associated with systolic blood pressure and pulse pressure. No significant associations were observed for PC2 (Table 6). These findings suggest that greater conversion of MMA to DMA may be associated with elevated blood pressure, consistent with results from the conventional and leave-one-out models.

The Spearman correlation between DMA% and arsenobetaine was 0.35, which suggests a moderate contribution from seafood intake to DMA, leaving inorganic arsenic exposure as the main contributor to DMA% in this study. In sensitivity analyses, the inclusion of the sum of methylated and inorganic arsenic in the models to adjust the models of arsenic metabolism for arsenic exposure did not appreciably change the results, nor did the inclusion of arsenobetaine (data not shown). Including alcohol consumption as a covariate in the fully adjusted models did not appreciably alter the effect estimates, indicating that the observed associations are robust to this additional adjustment (Supplementary Table 2). In linear regression models evaluating the interaction between eGFR and arsenic in each of the blood pressure outcomes, we did not observe evidence of statistical interaction (Supplementary Table 3), suggesting that kidney function within the range experienced by participants included in this analysis did not appreciably alter the relationship between biomarkers of urinary arsenic metabolism and blood pressure metrics.

## Discussion

In this cross-sectional study of adult men from El Salvador and Nicaragua, we observed that a higher relative proportion of urinary DMA was associated with adverse blood pressure traits, particularly elevated systolic blood pressure, pulse pressure, and mean arterial pressure. Total arsenic concentration was not associated with blood pressure endpoints. These findings suggest that there is a relationship between biomarkers of efficient arsenic metabolism and blood pressure endpoints, which are important cardiometabolic health outcomes.

Speciated arsenic analyses provide valuable insight into the differential toxicity of arsenic metabolites. Prior studies have shown that while both MMA and DMA are associated with adverse health outcomes, higher MMA% is more strongly associated with cancer risk, whereas higher DMA% is more strongly associated with cardiometabolic risk (32–36). A systematic review conducted by Kuo et al. reported mixed findings regarding the association between arsenic metabolites and cardiovascular outcomes (37). Some studies found that lower MMA% (and correspondingly higher DMA%) was associated with increased prevalence of hypertension (38–40), which is consistent with our study, while others reported the opposite (41–44). Our study is among the first to apply both leave-one-out and PCA modeling strategies to cardiometabolic outcomes. These approaches, which account for the interdependence of arsenic species, may offer a more nuanced understanding of the relationship between biomarkers of arsenic metabolism and various cardiometabolic health outcomes.

Though recent literature reviews generally support a relationship between total arsenic exposure and hypertension (11, 45, 46), individual studies have observed mixed results. In two studies conducted in the highly arsenic-exposed population of Bangladesh (median total urinary arsenic = 86 μg/L), one found a longitudinal relationship between urinary arsenic and increased systolic and diastolic blood pressure (47), while the other did not find a cross-sectional relationship between toenail arsenic exposure and either blood pressure metric (48). Our study population had moderate total urinary arsenic exposure (between 8 μg/L and 15 μg/L), and both longitudinal and cross-sectional studies with comparable exposure levels have observed mixed results (9, 10, 49–53). A review by Zhao et al. attributed these inconsistencies to differences in exposure profile, arsenic source, and study population (11). They also reported that the relationship between total arsenic exposure and hypertension exhibits nonmonotonicity (11), which is consistent with our observations. In a study conducted using data from the National Health and Nutrition Examination Survey (median urinary arsenic concentration = 8.3 μg/L compared to our median of 11.0 μg/L), there was no observed relationship between total arsenic or total arsenic minus arsenobetaine with blood pressure or odds of hypertension, however there was a moderate relationship observed between DMA concentration and hypertension odds (54). There has additionally been some epidemiological evidence for threshold effects. The Strong Heart Family Study found an association between total urinary arsenic exposure and blood pressure only at their most highly exposed study site (median urinary arsenic concentration = 14.1 μg/L), but not at study sites which exhibit more comparable urinary arsenic exposure to our study. Additionally, it may be the case that we do not observe a relationship between arsenic exposure (measured as concentration) and any of the tested cardiometabolic outcomes due to our exclusion of participants with a urinary arsenic level below 5 μg/L. This exclusion means that our referent group was not a low exposure group, therefore we observed an artificially limited distribution of arsenic exposures and the highly exposed participants differed less from the referent group than would have occurred in a study of all participants. Overall, our study is among the first to evaluate the relationship between arsenic exposure and blood pressure outcomes in a Central American population, which may contribute to discrepancies between our findings and existing literature.

Genetic variation plays a key role in arsenic metabolism. InAs is primarily metabolized by *AS3MT* and polymorphisms in the *AS3MT* gene can influence enzyme expression and the efficiency of arsenic methylation (55). Several variants in *AS3MT* have now been identified that are associated with arsenic methylation efficiency and the distribution of urinary arsenic metabolites (17, 56, 57). Interestingly, *AS3MT* was fine mapped in the Illumina Cardio Metabochip (an array including 200,000 SNPs) because genetic variants in this part of the genome (10q24) have been associated with blood pressure levels in non-targeted genome-wide association studies in general populations even in the absence of information on arsenic exposure (27, 59). Additionally, a Mendelian randomization trial using these variants to predict metabolic efficiency found that inefficient arsenic metabolism was overall associated with increased systolic and diastolic blood pressure among never smokers who consumed high levels of rice, known to be a prominent source of inorganic arsenic in many populations globally. This runs counter to our findings; however, their study did not directly quantify arsenic exposure, which limits the capacity for direct comparison.

The association that we found between efficient arsenic metabolism and an adverse health outcome is in alignment with other existing studies evaluating cardiometabolic health outcomes, though this finding runs counter to what is commonly understood with respect to cancer outcomes (37). The exact mechanism causing this relationship is unknown, but several possibilities have been posited (26, 60). One explanation is that, because the trivalent species of arsenic are more toxic than their pentavalent counterparts, the metabolic toxicity associated with efficient conversion to DMA is due to DMA^3+^, while the carcinogenesis of having a higher MMA proportion is related to an increased proportion of MMA^3+^. Measuring MMA^3+^ and DMA^3+^ is difficult in epidemiologic studies and it is thus hard to test these hypothesis. Because arsenic uses the one carbon metabolism (OCM) pathway, it is possible that the relationship is due to confounding by the essential nutrients related to the OCM pathway, such as choline, folate, or B vitamins. This possibility is supported by findings from the Strong Heart Family Study which found that a relationship between efficient arsenic metabolism and both HOMA-IR and waist circumference was attenuated after adjustment for OCM-related metabolites (60). However, a recent study which found a prospective association between efficient arsenic metabolism and metabolic syndrome observed no evidence of confounding by B vitamins (26).

The OCM pathway uses the enzyme SAM and various essential nutrients, including folate as a key element, giving folate a positive association with efficient arsenic metabolism (60). Folate additionally has an inverse relationship with blood pressure (61, 62). Because of this, folate may act as a negative confounder in the relationship between arsenic metabolism and blood pressure. Since it was unmeasured and not adjusted for, we expect that the effect estimates observed in this study could be biased toward the null. If we were able to adjust for folate, we would expect to see a stronger relationship between arsenic metabolism and blood pressure.

Additional physiological mechanisms may underlie the observed association between biomarkers of efficient arsenic metabolism and blood pressure traits. Arsenic exposure has been linked to increased calcium sensitization, decreased antioxidant defense mechanism, and increased myosin phosphorylation, all of which may contribute to higher blood pressure (55). Our findings suggest that these effects may be driven specifically by metabolic process which favors full conversion of InAs to DMA, either directly or through processes related to arsenic metabolism.

Reverse causation is often considered in cross-sectional studies evaluating the relationship between arsenic metabolism and cardiometabolic outcomes, and we cannot discard the possibility of reverse causation in this study. However, reverse causation mechanisms are often proposed to act through altered adiposity, which is statistically controlled for in this study through BMI, and is theorized to stem from alterations in estrogen production, which is a less relevant factor in our all-male population (63). Furthermore, longitudinal studies have shown a prospective association between arsenic exposure and hypertension, which do not support reverse causality as an explanation (37, 40, 64).

The MANOS study was initiated to investigate chronic kidney disease of unknown etiology (CKDu) in Central America (18). Although the relationship between blood pressure and CKDu is understudied, higher systolic blood pressure (particularly when diastolic blood pressure is maintained) has been identified as a risk factor for traditional chronic kidney disease (65–67). This pattern results in increased pulse pressure. These findings raise the possibility that arsenic exposure metabolism may contribute to kidney disease via its effects on systolic blood pressure.

This study has several limitations. First, because of the exclusion of participants with a total urinary arsenic concentration below 5 μg/L, we systematically exclude participants with low level arsenic exposure. This limitation likely did not affect our findings with arsenic metabolism proportions but may explain the null findings in our absolute concentration models because the referent group was not necessarily a low exposure group. Second, seafood and rice consumption is a direct source of DMA, therefore we could be concerned about capturing DMA exposure directly from fish or rice consumption, rather than as a metabolic product of arsenic metabolism (68, 69). This concern is mitigated by the moderate correlation between DMA% and arsenobetaine, which is also related to fish consumption, and by the fact that including arsenobetaine in the model did not appreciably change the results. Third, the relatively small sample size may have limited statistical power. Fourth, the cross-sectional design precludes the ability to establish temporality between arsenic exposure and blood pressure outcomes, however longitudinal studies have also observed this association (9, 47, 49). Fifth, blood pressure and arsenic metabolite measurements were both based on a single time point, which may not accurately reflect usual blood pressure status or relative arsenic metabolite levels. Additionally, because this is an occupational cohort, there is potential for the healthy worker bias, in which those who are most susceptible to arsenic toxicity may have self-selected out of employment due to poor health status. Because participants using antihypertensive medications or with blood pressure above 160/95 mmHg were excluded at enrollment, the study does not capture the full spectrum of blood pressure variation in the general population. This restriction could have biased our results toward the null by excluding individuals with the most adverse outcomes to arsenic exposure. Finally, we were unable to differentiate between oxidation states for each arsenic species in our analysis, which may have prevented us from seeing a relationship based on arsenic oxidation.

Despite these limitations, the study has several notable strengths. First, urinary arsenic metabolites are recognized as reliable biomarkers of exposure (70, 71). Second, the procedure for analyzing urinary arsenic metabolites was able to accurately detect and quantify >98% of arsenic species among all participants. Third, the use of three complementary modeling strategies enhances the robustness of the findings. Finally, the consistency of results across these approaches, along with sensitivity analyses, strengthens confidence in the observed associations.

## Conclusion

Our findings indicate that biomarkers of efficient arsenic methylation are strongly associated with adverse blood pressure outcomes. This suggests that individuals with higher arsenic methylation efficiency, reflected by greater conversion to DMA, may be at increased cardiovascular disease risk. This finding replicates existing literature on the relationship between arsenic metabolism and blood pressure in a novel study population. A key next step to investigating this relationship is to evaluate the longitudinal association between biomarkers of arsenic metabolism and blood pressure outcomes by determining both the prospective relationship between arsenic metabolites and blood pressure change overtime, and the prospective relationship between blood pressure and changes in arsenic metabolism overtime. Future research should also investigate the interplay between urinary arsenic species, *AS3MT* genetic variation, and blood pressure, as well as their potential contribution to the development of chronic kidney disease.

## Supplementary Material

Supplementary Files

This is a list of supplementary files associated with this preprint. Click to download.

• SupplementaryTables13.docx

## Figures and Tables

**Table 1.** Descriptive Statistics of the Participants in the MANOS Cohort.

Characteristic	El Salvador N = 182^1^	Nicaragua N = 211 ^1^	Full Cohort Before Exclusions N = 569^1^
**Age**	29 (14)	28 (10)	28 (12)
**BMI, kg/m^2^**	24.3 (5.3)	23.8 (4.7)	23.9 (5.0)
Missing			7
**Use Pesticides at Work**	15 (8.2%)	53 (25%)	104 (18%)
Missing			5
**Current Smoking Status**	69 (38%)	85 (40%)	154 (39%)
Missing			0
**Water Consumption at Work, L**	3.00 (1.54)	3.79 (3.08)	3.79 (2.50)
Missing			5
**Worksite**			
Sugar 1	47 (26%)	0 (0%)	51 (11%)
Sugar 2	15 (8.2%)	0 (0%)	16 (3.6%)
Sugar 3	0 (0%)	22 (10%)	22 (4.9%)
Sugar 4	0 (0%)	48 (23%)	50 (11%)
Construction	35 (19%)	0 (0%)	49 (11%)
Brick making	0 (0%)	89 (42%)	101 (22%)
Corn	85 (47%)	0 (0%)	106 (24%)
Plantain 1	0 (0%)	28 (13%)	29 (6.4%)
Plantain 2	0 (0%)	24 (11%)	26 (5.8%)
**Systolic Blood Pressure, mmHg**	124 (15)	125 (17)	124 (15)
Missing			4
**Diastolic Blood Pressure, mmHg**	75 (11)	74 (15)	73 (13)
Missing			4
**Pulse Pressure, mmHg**	50 (12)	50 (15)	51 (14)
Missing			4
**Summed Urinary Arsenic^2^, μg/L**	11.0 (11.2)	10.8 (10.7)	11.0 (10.9)
Missing			165
**Urinary InAs, %**	11.3 (6.3)	11.1 (6.2)	11.2 (6.2)
Missing			165
**Urinary MMA, %**	15.0 (5.7)	14.9 (5.4)	14.9 (5.6)
Missing			165
**Urinary DMA, %**	74.2 (10.1)	74.1 (10.2)	74.1 (10.0)
Missing			165
**Arsenobetaine, μg/L**	3.34 (6.75)	1.45 (4.27)	1.97 (5.15)
Missing			165

1 Median (IQR); n (%)

2 Summed urinary arsenic represents the sum of inorganic and methylated arsenic (InAs concentration + MMA concentration + DMA concentration)

**Table 2.** Conventional Modeling: Linear Regression Results of Relationship Between Concentration of Urinary Biomarkers of Arsenic Metabolism andBlood Pressure Metrics. All values in mmHG.

Model	Arsenic Component	Range	Systolic Blood Pressure		Diastolic Blood Pressure		Pulse Pressure		Mean Arterial Pressure	
			Coefficient	95% Confidence Interval	Coefficient	95% Confidence Interval	Coefficient	95% Confidence Interval	Coefficient	95% Confidence Interval
1	InAs	<0.78 μg/L (ref)								
		0.78–1.69 μg/L	−3.92	(−7.17, −0.67)	−2.36	(−4.91, 0.20)	−1.56	(−4.24, 1.11)	−2.88	(−5.39, −0.37)
		>1.69 μg/L	−2.35	(−5.90, 1.20)	−0.92	(−3.71, 1.88)	−1.44	(−4.36, 1.49)	−1.39	(−4.13, 1.34)
		P for Trend	0.20	0.52	0.34	0.32				
	MMA	<1.10 μg/L (ref)								
		1.10–2.02 μg/L	−4.31	(−7.49, −1.13)	−3.72	(−6.21, −1.24)	−0.59	(−3.21, 2.04)	−3.92	(−6.36, −1.48)
		>2.02 μg/L	−3.14	(−6.65, 0.36)	−1.14	(−3.88, 1.60)	−2.00	(−4.90, 0.892)	−1.81	(−4.50, 0.88)
		P for Trend	0.08		0.42		0.18		0.19	
	DMA	<2.72 μg/L (ref)								
		5.72–10.99 μg/L	−2.07	(−5.21, 1.06)	−2.42	(−4.87, 0.03)	0.35	(−2.23, 2.92)	−2.30	(−4.71, 0.11)
		>10.99 μg/L	−0.44	(−3.90, 3.01)	−0.19	(−2.89, 2.51)	−0.25	(−3.09, 2.58)	−0.28	(−2.93, 2.38)
		P for Trend	0.80		0.89		0.86		0.84	
	Total Arsenic	<7.82 μg/L (ref)								
		7.82–14.8 μg/L	−2.93	(−6.08, 0.22)	−2.56	(−5.03, −0.10)	−0.36	(−2.95, 2.23)	−2.69	(−5.11, −0.26)
		>14.8 μg/L	−1.50	(−4.99, 1.98)	−0.40	(−3.13, 2.32)	−1.10	(−3.96, 1.76)	−0.77	(−3.45, 1.91)
		P for Trend	0.40		0.77		0.45		0.57	
2	InAs	<0.78 μg/L (ref)								
		0.78–1.69 μg/L	−3.70	(−6.98, −0.43)	−2.51	(−5.08, 0.07)	−1.60	(−4.32, 1.11)	−2.64	(−5.13, −0.14)
		>1.69 μg/L	−1.85	(−5.42, 1.73)	−0.97	(−3.76, 1.83)	−1.58	(−4.55, 1.38)	−0.79	(−3.52, 1.94)
		P for Trend	0.31	0.85	0.30	0.57				
	MMA	<1.10 μg/L (ref)								
		1.10–2.02 μg/L	−4.10	(−7.28, −0.91)	−3.82	(−6.31, −1.33)	−0.63	(−3.27, 2.02)	−3.68	(−6.10, −1.26)
		>2.02 μg/L	−2.83	(−6.35, 0.68)	−1.18	(−3.92, 1.57)	−2.04	(−4.96, 0.88)	−1.47	(−4.14, 1.20)
		P for Trend	0.12		0.57		0.17		0.28	
	DMA	<5.72 μg/L (ref)								
		5.72–10.99 μg/L	−1.90	(−5.03, 1.22)	−2.47	(−4.92, −0.02)	0.29	(−2.30, 2.87)	−2.10	(−4.47, 0.28)
		>10.99 μg/L	−0.04	(−3.51, 3.43)	−0.22	(−2.93, 2.49)	−0.26	(−3.13, 2.61)	0.13	(−2.51, 2.77)
		P for Trend	0.98		0.87		0.86		0.92	
	Total Arsenic	<7.82 μg/L (ref)								
		7.82–14.8 μg/L	−2.81	(−5.95, 0.33)	−2.64	(−5.10, −0.17)	−0.41	(−3.01, 2.19)	−2.54	(−4.93, −0.15)
		>14.8 μg/L	−1.10	(−4.60, 2.39)	−0.44	(−3.17, 2.29)	−1.18	(−4.08, 1.71)	−0.32	(−2.97, 2.34)
		P for Trend	0.54		0.95		0.42		0.82	

Model 1: Models correct for osmolality, age, and BMI.

Model 2: All correct for the same covariates as Model 1, and add worksite, hydration, and work with agrochemicals.

**Table 3.** Conventional Modeling: Linear Regression Results of Relative Proportion of Biomarkers of Arsenic Metabolism and Blood Pressure Metrics. Allvalues in mmHg.

Model	Arsenic Component	Range	Systolic Blood Pressure		Diastolic Blood Pressure		Pulse Pressure		Mean Arterial Pressure	
			Coefficient	95% Confidence Interval	Coefficient	95% Confidence Interval	Coefficient	95% Confidence Interval	Coefficient	95% Confidence Interval
1	InAs	<9.12% (ref)								
		9.12%–13.13%	−0.94	(−4.04, 2.17)	0.55	(−1.82, 2.93)	−1.49	(−4.00, 1.02)	0.06	(−2.30, 2.41)
		>13.13%	−1.44	(−4.60, 1.72)	−0.64	(−3.06, 1.79)	−0.81	(−3.37, 1.75)	−0.90	(−3.31, 1.50)
		P for Trend	0.37		0.61		0.54		0.46	
	MMA	<12.39% (ref)								
		12.39%–16.07%	0.80	(−2.38, 3.98)	0.36	(−2.1, 2.82)	0.44	(−2.14, 3.02)	0.51	(−1.93, 2.94)
		>16.07%	−3.51	(−6.66, −0.36)	−0.69	(−3.12, 1.75)	−2.83	(−5.38, −0.27)	−1.63	(−4.04, 0.79)
		P for Trend	0.03		0.58		0.03		0.19	
	DMA	<71.28% (ref)								
		71.28%–77.51%	3.88	(0.96, 6.81)	1.32	(−0.94, 3.58)	2.56	(0.18, 4.94)	2.17	(−0.06, 4.41)
		>77.51%	3.77	(0.66, 6.88)	1.16	(−1.24, 3.57)	2.61	(0.08, 5.13)	2.03	(−0.34, 4.41)
		P for Trend	0.02		0.34		0.04		0.09	
2	InAs	<9.12% (ref)								
		9.12%–13.13%	−0.95	(−4.04, 2.14)	0.55	(−1.79, 2.88)	−1.50	(−4.01, 1.02)	0.05	(−2.28, 2.38)
		>13.13%	−1.42	(−4.58, 1.74)	−0.56	(−2.94, 1.83)	−0.86	(−3.43, 1.71)	−0.84	(−3.22, 1.54)
		P for Trend	0.38		0.65		0.51		0.49	
	MMA	<12.39% (ref)								
		12.39%–16.07%	0.61	(−2.57, 3.80)	−0.01	(−2.45, 2.43)	0.62	(−1.97, 3.22)	0.20	(−2.22, 2.61)
		>16.07%	−3.70	(−6.86, −0.55)	−0.94	(−3.36, 1.47)	−2.76	(−5.33, −0.19)	−1.86	(−4.26, 0.53)
		P for Trend	0.02		0.44		0.04		0.13	
	DMA	<71.28% (ref)								
		71.28%–77.51%	4.00	(1.06, 6.93)	1.36	(−0.89, 3.60)	2.64	(0.24, 5.04)	2.24	(0.01, 4.46)
		>77.51%	3.75	(0.65, 6.85)	1.18	(−1.18, 3.55)	2.57	(0.04, 5.10)	2.04	(−0.30, 4.39)
		P for Trend	0.02		0.33		0.05		0.09	

Model 1: Models correct for age, BMI, and worksite.

Model 2: All correct for the same covariates as Model 1, and add hydration, smoke, and work with agrochemicals.

**Table 4.** Leave One Out Modeling: Linear Regression of the Relative Proportion of Two Biomarkers of Arsenic Metabolism (Third Left Out) and Blood Pressure values in mmHg.

Model	Component Left Out	Arsenic Component	Range	Systolic Blood Pressure		Diastolic Blood Pressure		Pulse Pressure		Mean Arte
				Coefficient	95% Confidence Interval	Coefficient	95% Confidence Interval	Coefficient	95% Confidence Interval	Coefficient
1	InAs	MMA	<12.39% (ref)							
			12.39%–16.07%	0.92	(−2.66, 4.50)	0.54	(−2.24, 3.32)	0.38	(−2.53, 3.29)	0.67
			>16.07%	−2.10	(−6.73, 2.53)	0.27	(−3.33, 3.86)	−2.37	(−6.13, 1.39)	−0.52
			P for Trend	0.37		0.88		0.22		0.77
		DMA	<71.28% (ref)							
			71.28%–77.51%	2.32	(−1.25, 5.88)	1.25	(−1.51, 4.01)	1.07	(−1.83, 3.96)	1.61
			>77.51%	1.89	(−2.73, 6.51)	1.29	(−2.30, 4.87)	0.61	(−3.15, 4.36)	1.49
			P for Trend	0.42		0.48		0.75		0.41
	MMA	InAs	<9.12% (ref)							
			9.12%–13.13%	0.75	(−3.05, 4.55)	1.02	(−1.93, 3.97)	−0.27	(−3.35, 2.81)	0.93
			>13.13%	4.39	(−0.64, 9.43)	0.75	(−3.16, 4.66)	3.65	(−0.44, 7.73)	1.96
			P for Trend	0.09		0.71		0.08		0.32
		DMA	<71.28% (ref)							
			71.28%–77.51%	6.24	(2.50, 9.99)	1.32	(−1.58, 4.23)	4.92	(1.88, 7.95)	2.96
			>77.51%	7.23	(2.23, 12.2)	1.72	(−2.16, 5.60)	5.51	(1.46, 9.56)	3.56
			P for Trend	0.005		0.38		0.008		0.07
	DMA	InAs	<9.12% (ref)							
			9.12%–13.13%	−0.49	(−3.66, 2.67)	0.59	(−1.86, 3.05)	−1.09	(−3.65, 1.48)	0.23
			>13.13%	0.54	(−2.95, 4.03)	−0.34	(−3.05, 2.36)	0.88	(−1.94, 3.71)	−0.05
			P for Trend	0.76		0.80		0.54		0.97
		MMA	<12.39% (ref)							
			12.39%–16.07%	0.83	(−2.43, 4.10)	0.30	(−2.23, 2.83)	0.53	(−2.11, 3.18)	0.48
			>16.07%	−3.77	(−7.27, −0.28)	−0.51	(−3.21, 2.20)	−3.26	(−6.09, −0.43)	−1.59
			P for Trend	0.04		0.71		0.02		0.24
2	InAs	MMA	<12.39% (ref)							
			12.39%–16.07%	0.58	(−3.02, 4.18)	0.06	(−2.70, 2.81)	0.52	(−2.42, 3.46)	0.23
			>16.07%	−2.53	(−7.18, 2.11)	−0.19	(−3.75, 3.37)	−2.34	(−6.13, 1.45)	−0.97
			P for Trend	0.28		0.92		0.23		0.59
		DMA	<71.28% (ref)							
			71.28%–77.51%	2.35	(−1.21, 5.90)	1.22	(−1.50, 3.95)	1.12	(−1.78, 4.03)	1.60
			>77.51%	1.58	(−3.03, 6.20)	1.02	(−2.52, 4.55)	0.57	(−3.20, 4.33)	1.21
			P for Trend	0.50		0.57		0.77		0.50
	MMA	InAs	<9.12% (ref)							
			9.12%–13.13%	0.68	(−3.11, 4.48)	1.08	(−1.83, 3.99)	−0.39	(−3.49, 2.70)	0.95
			>13.13%	4.42	(−0.61, 9.46)	1.01	(−2.85, 4.87)	3.42	(−0.69, 7.52)	2.15
			P for Trend	0.09		0.61		0.10		0.27
		DMA	<71.28% (ref)							
			71.28%–77.51%	6.39	(2.65, 10.1)	1.48	(−1.38, 4.35)	4.91	(1.86, 7.96)	3.12
			>77.51%	7.24	(2.25, 12.2)	1.95	(−1.88, 5.77)	5.29	(1.22, 9.36)	3.71
			P for Trend	0.005		0.32		0.01		0.06
	DMA	InAs	<9.12% (ref)							
			9.12%–13.13%	−0.46	(−3.61, 2.70)	0.68	(−1.74, 3.09)	−1.13	(−3.70, 1.44)	0.30
			>13.13%	0.63	(−2.85, 4.11)	−0.15	(−2.81, 2.51)	0.78	(−2.05, 3.61)	0.11
			P for Trend	0.72		0.91		0.59		0.93
		MMA	<12.39% (ref)							
			12.39%–16.07%	0.63	(−2.65, 3.90)	−0.10	(−2.60, 2.41)	0.73	(−1.93, 3.39)	0.14
			>16.07%	−4.00	(−7.50, −0.50)	−0.85	(−3.53, 1.83)	−3.15	(−6.00, −0.30)	−1.90
			P for Trend	0.03		0.53		0.03		0.16

Model 1: InAs, MMA, and DMA models correct for age, BMI, and worksite.

Model 2: All correct for the same covariates as Model 1 and add hydration, smoking status and work with agrochemicals.

**Table 5.** Raw Loadings of Principal Components in PCA Analysis.

	PC1	PC2
InAs%	−0.875	−0.484
MMA%	−0.846	0.533
DMA%	0.999	0.027

**Table 6.** PCA Modeling: Linear Regression of PCs with Blood Pressure Metrics. All values in mmHg.

Model	Interpretation	Principal Component	Systolic Blood Pressure		Diastolic Blood Pressure		Pulse Pressure		Mean Arterial Pressure	
			Coefficient	95% Confidence Interval	Coefficient	95% Confidence Interval	Coefficient	95% Confidence Interval	Coefficient	95% Confidence Interval
1	Second Methylation Step	PC1	0.89	(0.06, 1.71)	0.14	(−0.49, 0.77)	0.74	(0.08, 1.41)	0.39	(−0.24, 1.02)
	First Methylation Step	PC2	−0.68	(−2.39, 1.04)	0.16	(−1.15, 1.48)	−0.84	(−2.22, 0.55)	−0.12	(−1.42, 1.19)
2	Second Methylation Step	PC1	0.93	(0.11, 1.75)	0.20	(−0.43, 0.82)	0.74	(0.07, 1.40)	0.44	(−0.18, 1.06)
	First Methylation Step	PC2	−0.79	(−2.50, 0.93)	−0.03	(−1.33, 1.27)	−0.76	(−2.15, 0.64)	−0.28	(−1.57, 1.01)

Model 1: InAs, MMA, and DMA models correct for age, worksite, and BMI.

Model 2: All correct for the same covariates as Model 1 and add hydration, smoking status, and work with agrochemicals.

## Data Availability

The datasets generated and/or analyzed during the current study are not publicly available due to privacy protections but are available from the corresponding author on reasonable request.

## List of Abbreviations

InAs: Inorganic Arsenic
AS3MT: Arsenite Methyltransferase
SAM: S-adenosyl methionine
MMA: Monomethylarsonous Acid
DMA: Dimethylarsinic Acid
MANOS: MesoAmerican Nephropathy Occupational Study
eGFR: estimated Glomerular Filtration Rate
BMI: Body Mass Index
MDL: Method Detection Limit
PCA: Principal Components Analysis
OCM: One carbon metabolism
CKDu: Chronic Kidney Disease of Unknown Etiology
